# Diversity and distribution of Myristicaceae in the Amazonian State of Acre, Brazil

**DOI:** 10.3897/BDJ.14.e196523

**Published:** 2026-08-06

**Authors:** Isadora Teles Lopes, Herison Medeiros, Rafaela Campostrini Forzza

**Affiliations:** 1 Universidade de São Paulo, São Paulo, Brazil Universidade de São Paulo São Paulo Brazil; 2 Jardim Botânico do Rio de Janeiro, Rio de Janeiro, Brazil Jardim Botânico do Rio de Janeiro Rio de Janeiro Brazil; 3 Universidade Federal do Oeste do Pará, Santarém, Brazil Universidade Federal do Oeste do Pará Santarém Brazil; 4 Instituto Chico Mendes de Conservação da Biodiversidade, Prado, Brazil Instituto Chico Mendes de Conservação da Biodiversidade Prado Brazil

**Keywords:** Amazon, nutmeg, sex ratio, species distribution, species richness, *ucuuba*, undersampled areas, watershed biodiversity.

## Abstract

**Background:**

The family Myristicaceae (Magnoliales) comprises ca. 500 species and has a pantropical distribution and centres of diversity in Africa, Asia and the Neotropics. In the latter, the Amazon Region, the largest rainforest in the world, is notable for having about 65 Myristicaceae species and recorded hyperdominance with 29 species of five genera, with a highlight for *Iryanthera* Warb. and *Virola* Aubl. genera with 13 and 11 hyperdominant species, respectively. Although the Amazon is known to be highly biodiverse, with 14,003 known seed plant species, much remains unknown due to the lack of proper research funding and difficulty in accessing many areas. This study aims to increase our knowledge of the family and the region by providing curated data about the occurrence of Myristicaceae species in the Amazonian State of Acre in Brazil.

**New information:**

We provide a dataset with 34 species and five genera of Myristicaceae recorded in Acre, Brazil, treated in the most recent flora by the authors. This includes the nine new occurrences for the State and corresponds to ~ 31% of the family’s species richness in the American continent. The distributions exhibit different patterns, with 16 species found throughout the State and 18 species restricted to one of two watersheds. This difference between the diversity in the two basins was previously observed in other studies of birds and vines and here which we observed may also be the case for trees and treelets. We also observed the ratio of staminate and pistillate inflorescences amongst the collections, with a staminate-biased ratio of approximately 7:1.

## Introduction

The second largest family of order Magnoliales is Myristicaceae (APG IV (Angiosperm Phylogenetic Group) 2016), with around 500 species, 21 genera and a pantropical distribution in Africa, Asia and the Neotropics (WFO 2025). In the American continent, there are six genera and around 110 species (Kühn and Kubitzki 1993, Helmstetter et al. 2024), including approximately 65 known species in the Amazon rainforest (Jaramillo-Vivanco and Balslev 2020, Santamaría-Aguilar and Lagomarsino 2022, Zárate-Gómez 2022, Flora e Funga do Brasil 2025). In Brazil, the family is represented by 64 species (12 endemic) and, besides the five species found exclusively in the Cerrado or Atlantic Forest, most are found in Amazonia (Flora e Funga do Brasil 2025). In the Brazilian Amazon, they mainly occur in terra firme forest, *igapó* and *várzea* (inundated forests) and *campinarana* (white sand forests with poor soil; Rodrigues (1980), Flora e Funga do Brasil (2025)). Although the family corresponds to ~ 1% of the Amazonian tree species diversity, hyperdominance has been registered for 29 species of five genera, ~ 45% of the species recorded in the Amazon, highlighting the importance of species for the forest structure and dynamic in the domain (ter Steege et al. 2013, Draper et al. 2021).

The Amazon is the largest rainforest in the world, extending over 6 million km², has 14,003 known plant species from lowland forests (of which 6,727 are trees) and is the most important source of biodiversity for the Neotropics and around half of Brazil’s territory (Cardoso et al. 2017, Antonelli et al. 2018, IBGE - Instituto Brasileiro de Geografia e Estatística 2024). These numbers were estimated up to 10,071 tree species (ter Steege et al. 2019) and although we mentioned the 6,727 due the specialists taxonomic identification, we agree the number of tree species has already been surpassed, for example, considering more recent numbers for the Brazilian Amazon with 4,906 (Flora e Funga do Brasil 2026), compared to the previous count with 4,539 (Cardoso et al. 2017).

Despite the high known and estimated species richness, part of the known flora still is poorly understood and/or sampled, resulting in knowledge gaps about the biodiversity in the domain. This problem is mostly due to how difficult it is to access many areas and a lack of investing in science and research in the Amazon Region (Cardoso et al. 2017, Hopkins 2019). Compared to the Atlantic Forest, the number of known species in Amazonia is smaller (Flora e Funga do Brasil 2025), even though it occupies a larger area (~ 6.7 million km² vs. ~ 1.4 million km²). Anthropogenic fires and constant deforestation in the Amazon Region are also notable, which are aggravating factors for both the known and unknown flora, increasing the risk for many species and loss of biodiversity (Stropp et al. 2020). In 2024 alone, almost 6,300 km² of forest were destroyed and, although since 2023, the deforestation rates have decreased, thousands of kilometres have been consistently destroyed per year for decades (INPE – Instituto Nacional de Pesquisas Espaciais. Amazônia Legal-Prodes (Desmatamento) 2024).

In 2008, the first catalogue of the flora of Acre, Brazil (Daly and Silveira 2008) was published. This was an important study conducted by the *Universidade Federal do Acre* (UFAC) and the New York Botanical Garden (NYBG) and the only study focused on an entire Amazonian State to this day. The authors identified 28 Myristicaceae species. However, according to Medeiros et al. (2014), more plant species have been recorded in the State since then, which raised the question if this is the case for the family. The Flora e Funga do Brasil (2023) added six Myristicaceae species for the State and online surveys in databases also suggested there are over 30 species. The goal of this study was to provide a properly analysed and curated dataset of Myristicaceae specimens collected in Acre using a recent floristic study of the family in the State (Lopes et al. 2025).

## Project description

### Design description

The State of Acre is in the North Region of Brazil in south-western Amazonia, has an area of 164,000 km² (ranging from 07°07 to 11°08S latitude and 66°30 to 74°W longitude), lies completely within the Amazonian domain and has the following vegetation phytophysionomies: open ombrophilous forest, dense ombrophilous florest, dense submontane forest, alluvial or floodplain forests and formations on white sand known as *campinaranas*. The State is drained by two watersheds: the Juruá Basin in the western part and the Purus Basin in the eastern part (Daly and Silveira 2008, Acre. Governo do Estado do Acre 2010, Medeiros et al. 2014, IBGE - Instituto Brasileiro de Geografia e Estatística 2024).

## Sampling methods

### Sampling description

To analyse the richness and distribution of Myristicaceae species in Acre, data about specimens collected in the State were acquired from the online databases GBIF (2022), speciesLink (2025), Reflora – Herbário Virtual (2025), JABOT (2025) and manually compiled into an Excel sheet. Collections deposited in the Herbaria B, CEN, F, HUEFS, IAN, INPA, K, M, MBM, MICH, MIRR, MO, NL, NY, P, RB, SPF, UFACPZ, US and W (Thiers 2023) were reviewed in person or online during the flora study of the family for the State (Lopes et al. 2025). Specimens properly identified were included in the study, duplicates were identified during the compiling proccess and only one collection per specimen was selected for the dataset, the other duplicates being excluded. The specimens consulted online that could not be identified or confirmed, based on a previous identification, either because of low image resolution or the need for further analysis of the vegetative and reproductive structures, were not included. Specimens that were wrongly input into the databases as Myristicaceae and/or from other States were discarded.

The species distributions were based on the geographic coordinates on the exsiccata labels; every occurrence point was checked individually with the assistance of Google Earth (2025) and compared with the described locality. When the coordinates were unavailable, either not recorded or erroneous, the locality description was used to infer the coordinates with the tool geoLoc (2023), that provides coordinates of registered collections in speciesLink by searching the locality and Google Earth (2025), to find the described area. Misplaced information in the locality description was omitted (e.g. habitat and specimen descriptions). The specimens were mapped using the coordinates organised in an Excel sheet, QGIS v.3.40 (QGIS – Quantum GIS Development Team 2024) and the following base maps: Brazilian federal units, land coverage and use for the State of Acre and the national hydrographic division made available by the *Instituto Brasileiro de Geografia e Estatística* (IBGE - Instituto Brasileiro de Geografia e Estatística 2024).

## Geographic coverage

### Description

The geographic coverage encompasses the State of Acre in the Brazilian Amazon, located in the extreme west of the country, between the coordinates 07°07 to 11°08 S and 66°30 to 74°W.

## Taxonomic coverage

### Description

Our compiled dataset included 2,642 raw inputs from the online databases, resulting in 405 curated records of Myristicaceae from Acre.

**Figure 1.**
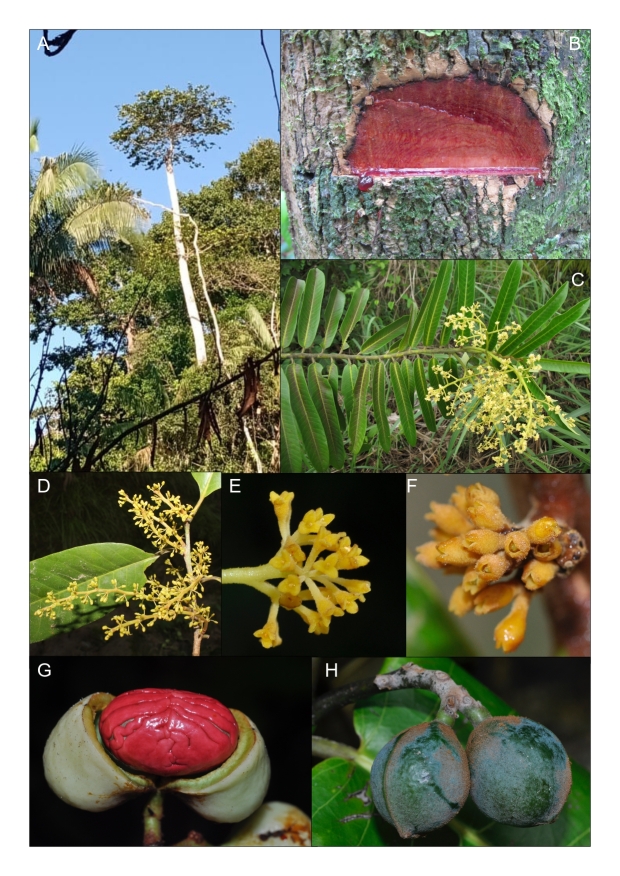
Vegetative and reproductive aspects of Myristicaceae. **A** *Virola surinamensis*, habit; **B** *Iryanthera juruensis*, longitudinal trunk cut showing red sap; **C** *V. surinamensis*, branch with leaves and inflorescence; **D** *Otoba parvifolia*, leaf and inflorescence; **E** *V. pavonis*, close-up view of staminate flowers; **F**. *V. peruviana*, close-up view of pistillate flowers; **G**. *I. juruensis*, fruit and seed with red aril; **H**. *V. peruviana*, fruits.

Myristicaceae are trees and shrubs, usually with a red watery sap and dioecious or rarely monoecious. They have alternate, simple leaves, paniculate or fasciculate-racemose inflorescences, small unisexual flowers with a uniseriate perianth and dehiscent and fleshy fruits that split into two halves, with a single seed enveloped by a fleshy aril (Fig. 1)

We recorded the occurrence of 34 species and five genera (Table 1): *Compsoneura sprucei* (A.DC.) Warb., *C. ulei* Warb., *Iryanthera crassifolia* A.C.Sm., *I. elliptica* Ducke, *I. juruensis* Warb., *I. laevis* Markgr., *I. lancifolia* Ducke, *I. macrophylla* (Benth.) Warb., *I. olacoides* A.C.Sm., *I. paradoxa* (Schwacke) Warb., *I. paraensis* Huber, *I. sagotiana* (Benth.) Warb., *I. tricornis* Ducke, *I. ulei* Warb., *Osteophloeum platyspermum* (Spruce ex A.DC.) Warb., *Otoba glycycarpa* (Ducke) W.A.Rodrigues & T.S.Jaram., *O. parvifolia* (Markgr.) A.H.Gentry, *Virola albidiflora* Ducke, *V. calophylla* Warb., *V. carinata* (Benth.) Warb., *V. decorticans* Ducke, *V. duckei* A.C.Sm., *V. elongata* (Benth.) Warb., *V. excisa* D.Santam., *V. flexuosa* A.C.Sm., *V. loretensis* A.C.Sm., *V. marleneae* W.A.Rodrigues, *V. mollissima* (Poepp. ex A.DC.) Warb., *V. pavonis* (A.DC.) A.C.Sm., *V. peruviana* (A.DC.) Warb., *V. rugulosa* Warb., *V. sebifera* Aubl., *V. surinamensis* (Rol. ex Rottb.) Warb. and *V. yasuniana* D.Santam.

**Figure 2.**
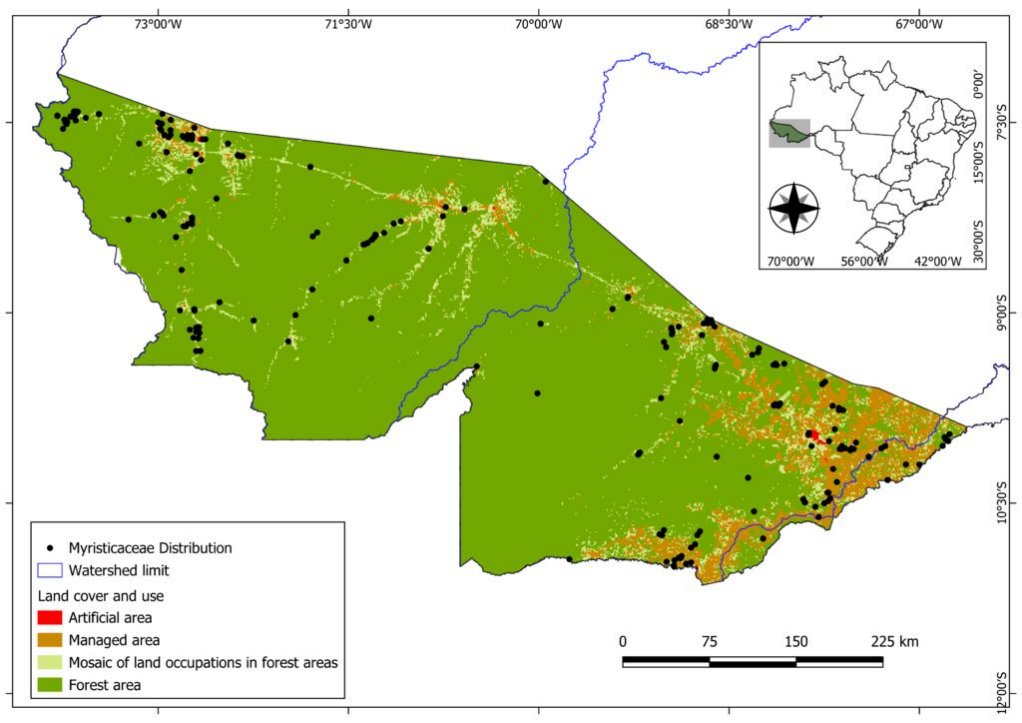
Distribution of the studied collections of Myristicaceae species in the State of Acre, showing specimens collected closer to urban areas, roadsides and river corridors.

All species were found mainly in vegetation in urban areas and their surroundings (46 records), on roadsides (173), close to rivers (140) or close to both roadsides and rivers (27, Fig. 2). Around 16 specimens were collected in Conservation Units (CUs) and are close to rivers, but were probably accessed through smaller dirt roads. We found 115 specimens collected in CUs and 16 collected in Indigenous Territory (IT), both demarcated and/or recognised by federal, State and municipal governments and observed that CUs with local bases of federal institutions associated with the Ministry of the Environment enabled a better planned and structured research expedition.

This collection pattern is probably caused because it is easier to access and collect in the mentioned areas, as well as transport the collected specimens, resulting in a higher number of records from these places. This is associated with what Cardoso et al. (2017) and Hopkins (2019) discuss about undersampling in the Amazon and how the proportionally few studies in the region still suffer because it is difficult to access many areas. These dificulties may result in a spatial bias, with collections concentrated in few areas; here, we observed municipalities with large land areas with fewer records than smaller ones (Table 2). The clustered collections can be related to area accessibility, but also sampling effort; for example, the higher number for the municipalities *Mâncio Lima* and *Cruzeiro do Sul* are linked to expeditions, flora projects destinations and CUs cataloguing.

Nevertheless, we were able to identify a significant diversity in these areas and an improvement since the first result for the flora of the State (Daly and Silveira 2008). Furthermore, we highlight the importance of research in this region. Even with the acknowledged limitations, the number of species recorded for this family in the State has been reviewed and increased: two new *Virola* species described by Santamaría-Aguilar and Lagomarsino (2022) were identified amongst multiple specimens previously collected and a few species that had not been collected for over a decade were collected again in the last five years of the dataset. Thus, the continued study of the flora of Acre has provided important results, as stated by Medeiros et al. (2014).

The best documented species are *Iryanthera juruensis* (75 specimens, Fig. 3b), *Virola elongata* (48, Fig. 4b), *V. sebifera* (35, Fig. 4d), *Otoba parvifolia* (32, Fig. 3e) and *V. calophylla* (25, Fig. 3f). All five are widely distributed throughout the State, have been recorded in every decade since the 1970s and were also collected with staminate and pistillate inflorescences and fruits, except for pistillate inflorescences of *O. parvifolia* that have not been registered for the State. Conversely, some species are notable for being undersampled: 13 species have less than five records and *I. olacoides*, *I. tricornis*, *V. marleneae* and *V. rugulosa* are represented by only one record.

Besides the five species mentioned earlier, another 11 are also broadly distributed; even with fewer specimens to analyse, we were able to identify occurrences in different areas of the State, totalling 47% of the species in this study. The other 18 species were shown to have restricted distributions and occur exclusively in one of the two watersheds in the State, including 16 species in the Juruá Basin (47%) and two in the Purus Basin (6%). This pattern of different species occurring in each watershed was previously observed by Guilherme (2012) for birds and Medeiros et al. (2016) for vines and lianas (*Paullinia* L., Sapindaceae) and the distributions observed here for Myristicaceae species suggest that these differences might also be the case for trees and treelets (Table 3). Amongst the 405 records, we estimate 241 specimens were collected in the Juruá Basin and 164 in the Purus Basin, ca. 59% and 41% of the records of restricted species, respectively. The ratio of broad and restricted distributions is close to the one found by Medeiros et al. (2016), mentioned as ca. 60% of species associated with watersheds, but the richness ratio in each basin was very different, ca. 29% in Juruá Basin and 31% in Purus Basin vs. here with 47% and 6%. Guilherme (2012) has also observed a higher species richness associated with the Juruá Basin; however, the ratio was not as disparate, with 16% and 5%, respectively. While the records examined here were duly curated and treated, we highlight the need for continued sampling efforts in the region to corroborate and/or adjust the patterns observed, which will allow more robust and representative spatial mapping of species.

## Traits coverage

**Sex ratio**

Due to the dioecious character of most Myristicaceae species, the majority of the collected specimens have only an inflorescence or infructescence (Table 4), with a few exceptions amongst *Iryanthera* species and *Virola peruviana* that have staminate and pistillate inflorescences or an infructescence and one or both inflorescences on the same collection. Previous studies, focusing on the ecology of the family, have registered the ratio of individuals producing staminate and pistillate flowers often being staminate-biased and a higher production of staminate flowers in the overall time of the studies (Armstrong and Irvine 1989, Ackerly et al. 2009, Queenborough et al. 2013). Although here we do not focus on the ecological aspects of Myristicaceae, we noticed a ratio of 135:20 regarding inflorescences found amongst the exsiccatae. However, the number of specimens collected while fruiting is higher than those collected while flowering (214 vs. 160) and this shows that, even with the biased ratio, there were multiple pistillate flowers successfully pollinated.

## Temporal coverage

### Notes

**Figure 5.**
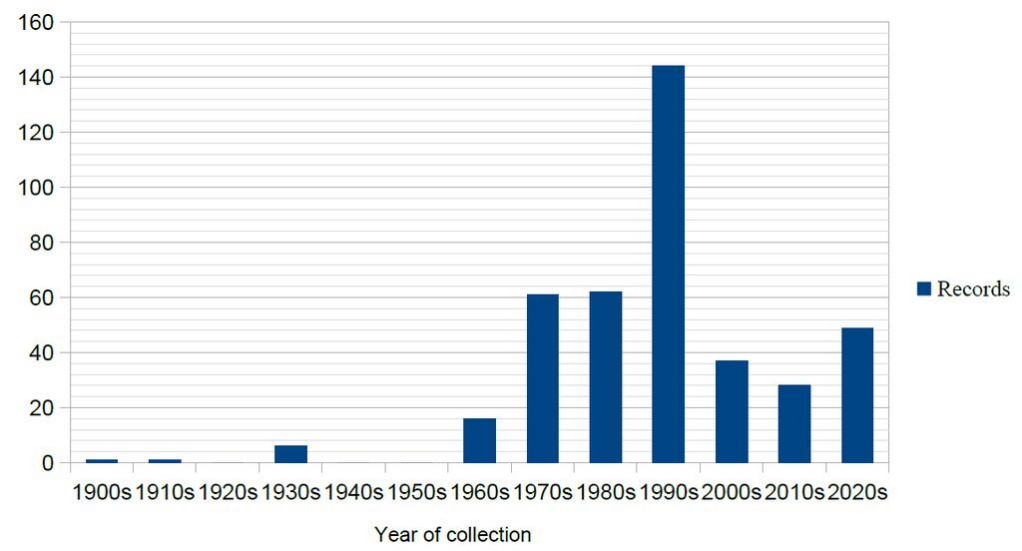
Graph of Myristicaceae collections in Acre across the decades, starting with the specimen collected during E.H.G. Ule's pioneer expedition to the Amazon, with an increase after the 1960 due projects focused on documenting the Amazonian flora and a peak during the 1990s when the partnership between UFAC and NYBG to catalogue the flora of Acre started.

The records date from 1901 to 2023 (122 years, Fig. 5), with more consistent collections from 1966 onwards.

Until 1950, the period of the pioneers (Daly and Silveira 2008), we have registered only three collectors for the family: Ernst H. G. Ule, Boris Krukoff, both European botanists who undertook expeditions to the Amazon Region and Adolpho Ducke, botanist and entomologist who studied the Amazon flora for almost 60 years. These collections are well conserved, making the taxonomic analysis reliable, but, regarding the locations, the reliability was mixed. For fixed places, such as “near mouth of *Rio* (river) *Macauhan*, tributary of the *Rio Iaco*”, we could infer the coordinates, but others, such as “*Rio Juruá*”, referring to a river with an extension of roughly 3,350 km, were considered dubious and removed from the mapping process. The first collection of Myristicaceae in Acre was done during Ernst Ule’s pioneer expedition, was analysed at the RB Herbarium (Lopes et al. 2025), but is also deposited in H, HGB, K, L and MO Herbaria and is cited as the first specimen collected of *Iryanthera ulei*.

The years between 1966 and 1987 were marked by large projects to study the Amazon flora, such as Plants of the Brazilian Amazonia, *Projeto RADAMBRASIL* (Radar in the Amazon Project) and *Projeto Flora Amazônica - PFA* (Amazonian Flora Project) and feature 125 collections treated in this research.

The decade with the most records (144) is the 1990s. This number is related to the partnership between UFAC and NYBG for the Floristics and Economic Botany of Acre project, which focused on documenting the Acre flora and resulted in a growth of 500% of the UFACPZ Herbarium, starting at ca. 5,000 collections and reaching 30,000 almost 20 years later (Daly and Silveira 2008).

In more recent years, in the 2020s, the number of collections increased compared to the two previous decades thanks to a scientific expedition by the “Monitora Programme” of the *Instituto Chico Mendes de Conservação da Biodiversidade* (*Programa Nacional de Monitoramento da Biodiversidade*; https://www.gov.br/icmbio/pt-br/assuntos/monitoramento; Cronemberger et al. (2023)), that focused on biodiversity monitoring and continued conservation assessment, travelling to the Conservation Unit *Parque Nacional da Serra do Divisor*, bordering Peru; and also due the flora study of the family in the State (Lopes et al. 2025).

**Figure 6.**
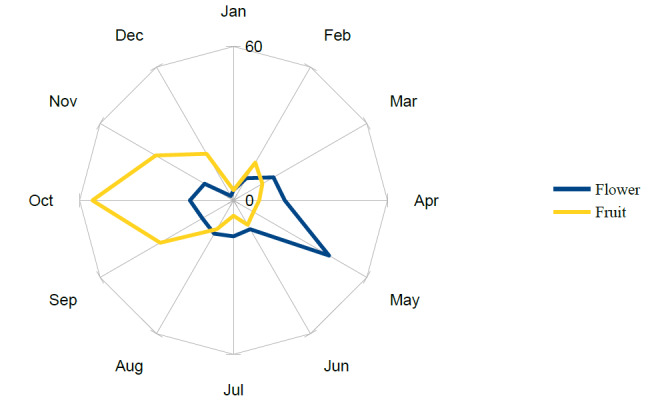
Graph of records of flowering and fruiting specimens per month, with records in almost every month and a peak of flowering specimens in May and another of fruiting specimens in October.

Specimens were collected during all months of the year, notably May for flowering specimens and October for fruiting specimens (Fig. 6). Compared to phenological studies of the species *Osteophloeum platyspermum* and *Virola michelii* Heckel (Alencar et al. 1979; Aleixo et al. 2023), different months of flowering and fruiting peaks were registered, but flowering peaks tended to be just before the wet season, during the transitional period between the dry and wet seasons and fruiting peaks tended to be during the wet season. Here, we found a similar pattern, with May representing a transitional period and October the start of the wet season (Embrapa Acre 2012). While the Amazon Forest houses extensive levels of rainfall thoughout the year, there are periods of more or less water volume and although it is best to analyse each species individually, due to phenological characteristics and differences of each one, this result suggests that flowers may be favoured by less rainfall in comparison to fruits.

## Usage licence

### Usage licence

Other

## Data resources

### Data package title

Distribution of Myristicaceae species in Acre, Brazil

### Resource link

https://doi.org/10.5281/zenodo.19672027

### Number of data sets

1

### Data set 1.

#### Data set name

Distribution of Myristicaceae species in Acre, Brazil

#### Data format

CSV

#### Character set

UTF-8

#### Description

Dataset of Myristicaceae species in the State of Acre, Brazil. It contains 405 records for the 34 species. The dataset was built by compiling data from four databases and analysed herbarium collections; it contains information on the taxonomy and distribution of the species and reproductive structures collected.

The specimens were identified mostly by W.A. Rodrigues, a specialist who researched the family for decades and I.T. Lopes during the flora study of the family in the State (Lopes et al. 2025). The collectors that contributed the most were D.C. Daly (60 records), C.A. Cid Ferreira (57), I.T. Lopes (34), G.T. Prance (31) and H. Medeiros (25), all related to projects of documenting the Acre flora. The herbaria with larger numbers of records are NY (208 records), UFACPZ (199), INPA (178) and RB (142). The Herbarium UFACPZ is located in the State capital, Rio Branco, where it is important to deposit collections of the local flora. The NY Herbarium houses many specimens due the partnership for the Flora of Acre project, many duplicates being sent to the institution. INPA is the largest herbarium in the Brazilian Amazon, with not only collections of multiple states, but also many of the first collected specimens in the Amazon Forest. Additionally and lastly, RB is the largest Brazilian herbarium and one of the largest in all Latin America, with over 900,000 collections and is a partner in multiple Amazonia focused research and expeditions.

**Data set 1.** 

Column label	Column description
occurrenceID	Sequencial number of treated collections.
kingdom	Name of the kingdom in which the taxon is classified.
phylum	Name of the phylum in which the taxon is classified.
class	Name of the class in which the taxon is classified.
order	Name of the order in which the taxon is classified.
family	Name of the family in which the taxon is classified.
genus	Name of the genus in which the taxon is classified.
specificEpithet	Taxon specific epithet.
scientificNameAuthorship	Author of the species.
scientificName	Full scientific name with author.
taxonRank	The hierarchical level in which the taxon is taxonomic classified, such as species, genus, family etc.
institutionCode	Acronym of the herbarium (according to Thiers 2023) where the selected collection was deposited.
catalogNumber	Herbarium voucher code.
otherCatalogNumbers	Herbarium voucher barcode.
basisOfRecord	The specific nature of the data record.
recordedBy	Collector of the specimen.
recordNumber	Collector’s number for the specimen.
year	Year when the specimen was collected.
month	Month when the specimen was collected.
day	Day when the specimen was collected.
eventDate	Date when the specimen was collected in the format YYYY-MM-DD.
country	Country where the specimen was collected.
stateProvince	Brazilian State where the specimen was collected.
municipality	Municipality where the specimen was collected.
locality	Detailed information about the location where the specimen was collected.
decimalLatitude	Record latitude in decimal format.
decimalLongitude	Record longitude in decimal format.
geodeticDatum	Coordinate system used: WGS84.
occurrenceRemarks	Which reproductive structure was found in the analysed collection or lack of.

## Additional information

**Conclusion**

The dataset presented here aims to provide reliable information and contribute to what is known about Myristicaceae and the Brazilian and Amazon flora and it includes 34 species recorded for Acre State. Acre has high Myristicaceae diversity, with ~ 31% of the species recorded in the American continent occurring in the State and new occurrences since previous floristic studies. Results for some species with fewer records suggest that even with the increased data, there are probably undersampling issues, linked to the knowledge gaps associated with the Amazon Region. The mapping proccess made evident clustered collections, close to areas of easier access and collection peaks caused by projects and expeditions focused on cataloguing the Amazonian and Acre flora and this curated dataset can be used to discuss undersampled areas, targeted efforts and their results.

Future collections of specimens could provide information for more robust distribution mapping, but with the current data, we were able to identify species with broad and restricted distributions and differences in the diversity between watersheds, a pattern previously noted in other studies that could also be the case for trees and treelets. These observed patterns can contribute to a better understanding of species distribution in the Amazon Region and watershed influence on biodiversity, especially in the State of Acre.

We also aim to support previous statements that addressed the importance of research and conservation initiatives in the Amazon Region. For example, in the 3 years in the 2020s included in this study, more collections of Myristicaceae were made than in the previous decade and multiple specimens were collected during a scientific expedition by the “Monitora Programme” and the flora study of the family in the State. These efforts not only contributed to the knowledge of the species occurring in Acre, but to Myristicaceae as a whole and the flora protected by Conservation Units. This dataset can also contribute to future floristic studies, such as for the State or southwest region of Amazonia, for example. Therefore, we want to reiterate how investing in studies and conservation has provided important results, even with the acknowledged difficulties in the region.

## Figures and Tables

**Figure 3a.**
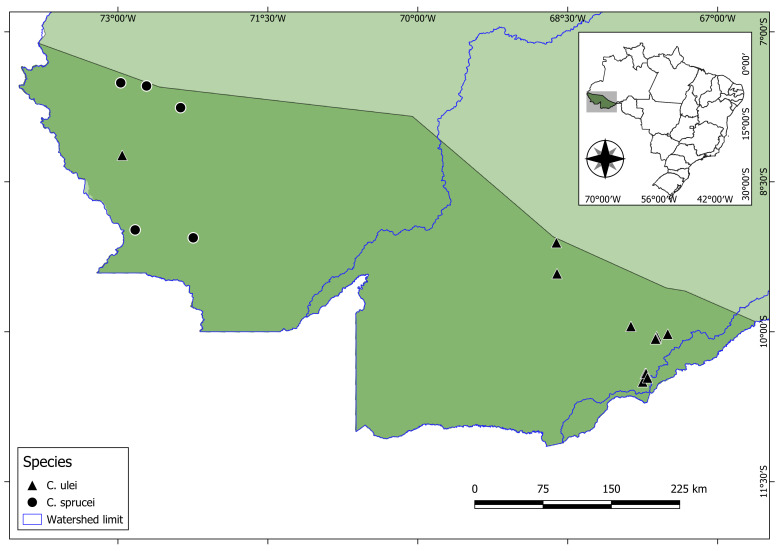
*Compsoneura sprucei* and *C. ulei*;

**Figure 3b.**
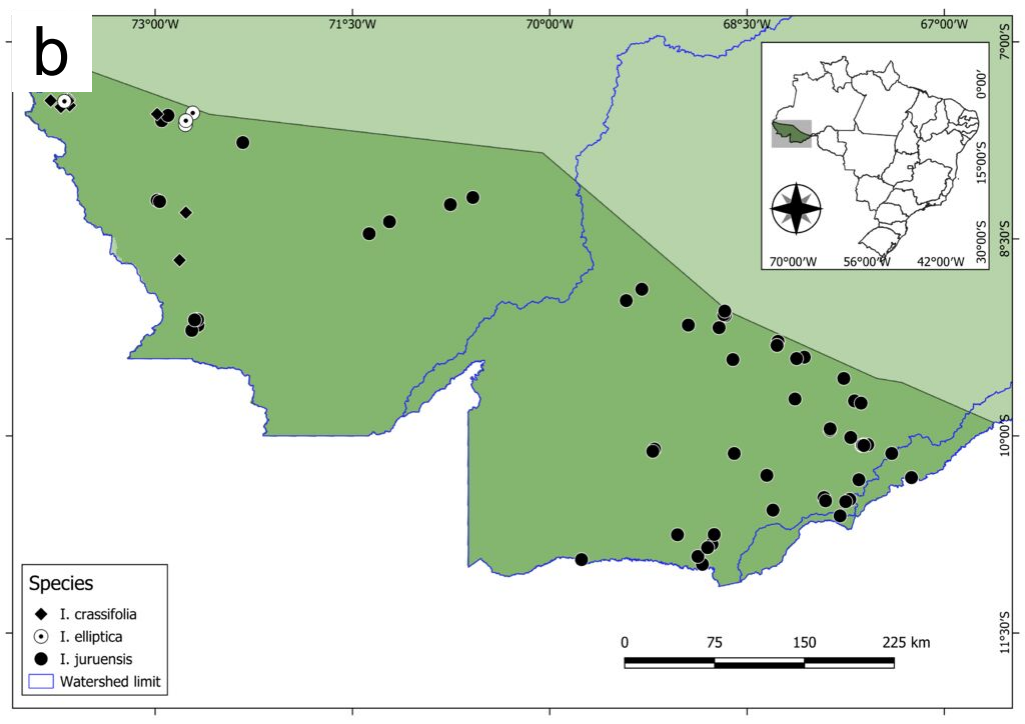
*Iryanthera crassifolia*, *I. elliptica* and *I. juruensis*;

**Figure 3c.**
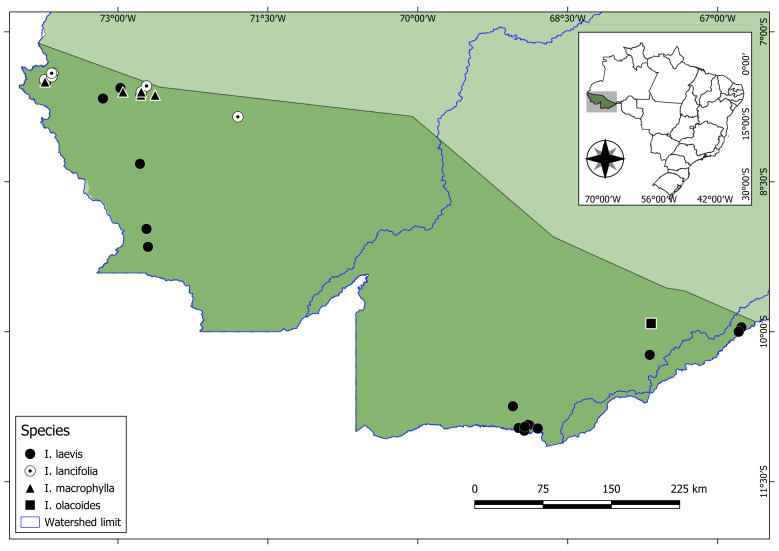
*Iryanthera laevis*, *I. lancifolia*, *I. macrophylla* and *I. olacoides*;

**Figure 3d.**
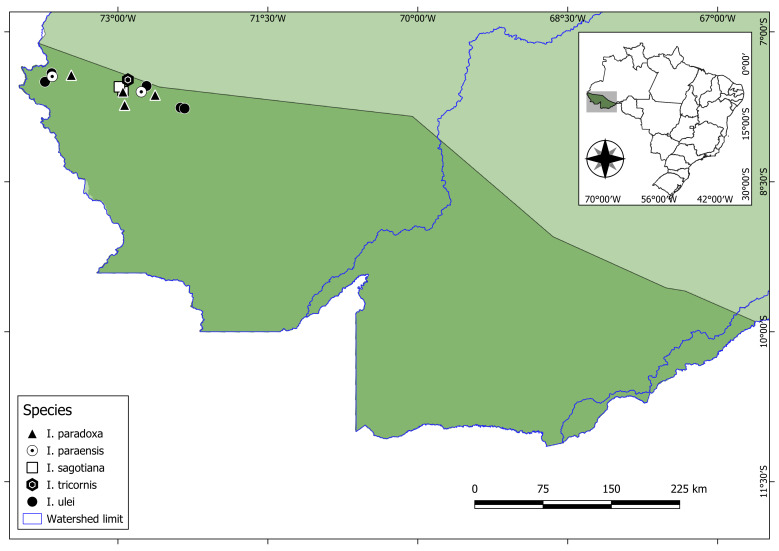
*Iryanthera paradoxa*, *I. paraensis*, *I. sagotiana*, *I. tricornis* and *I. ulei*;

**Figure 3e.**
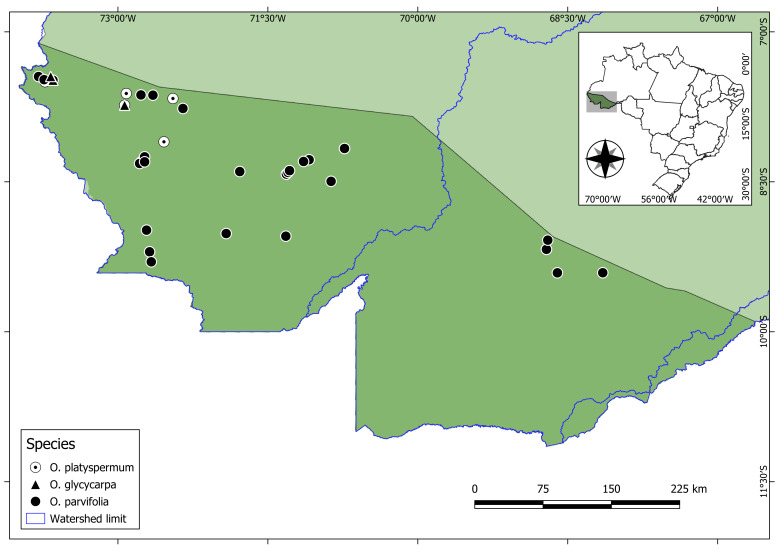
*Osteophloeum platyspermum, Otoba glycycarpa* and *O. parvifolia*;

**Figure 3f.**
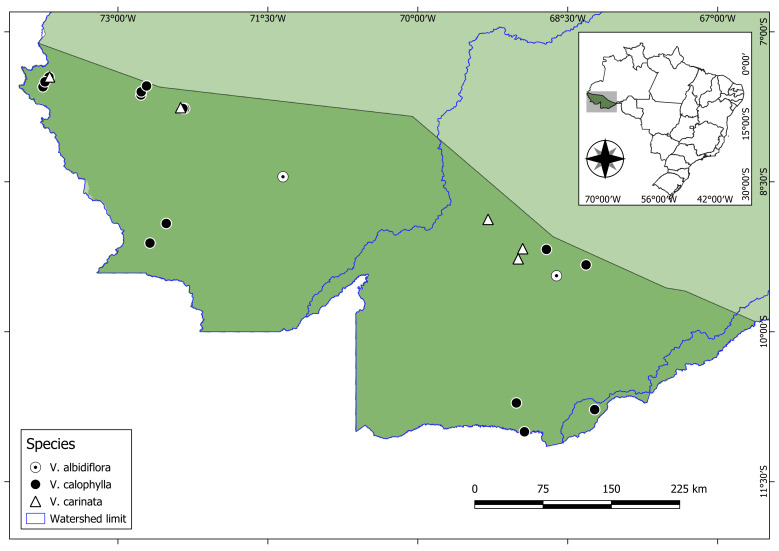
*Virola albidiflora, V. calophylla* and *V. carinata*.

**Figure 4a.**
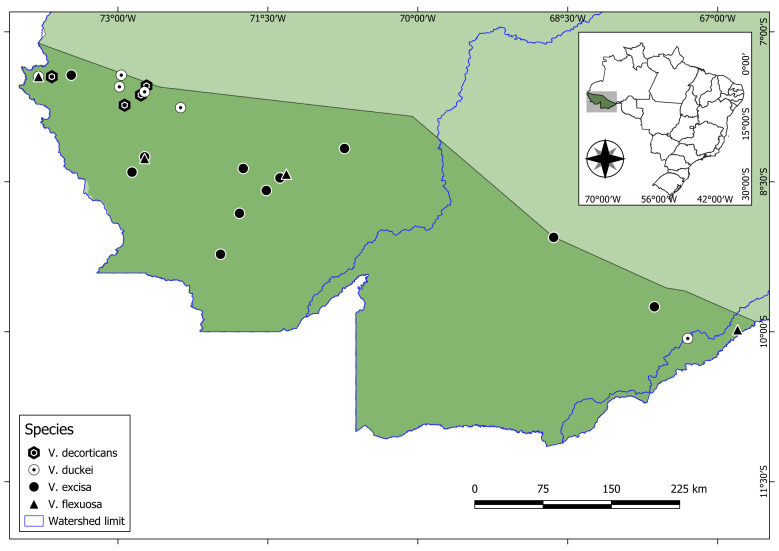
*Virola decorticans, V. duckei*, *V. excisa* and *V. flexuosa*;

**Figure 4b.**
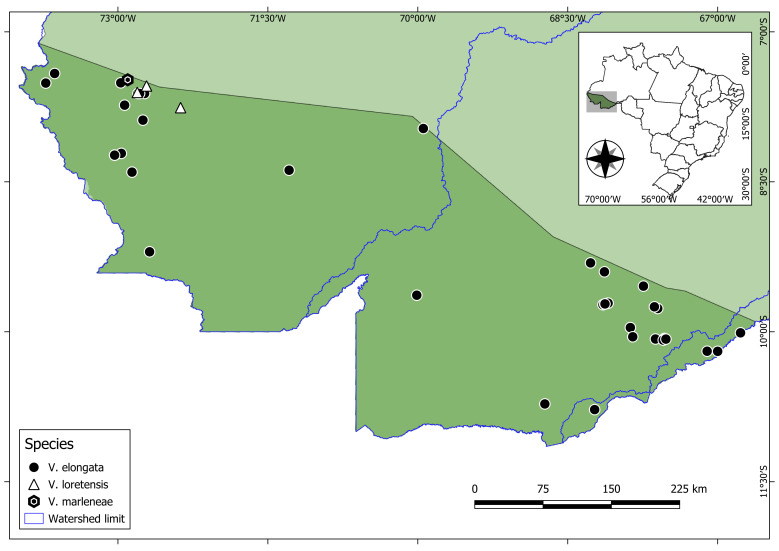
*Virola elongata*, *V. loretensis* and *V. marleneae*;

**Figure 4c.**
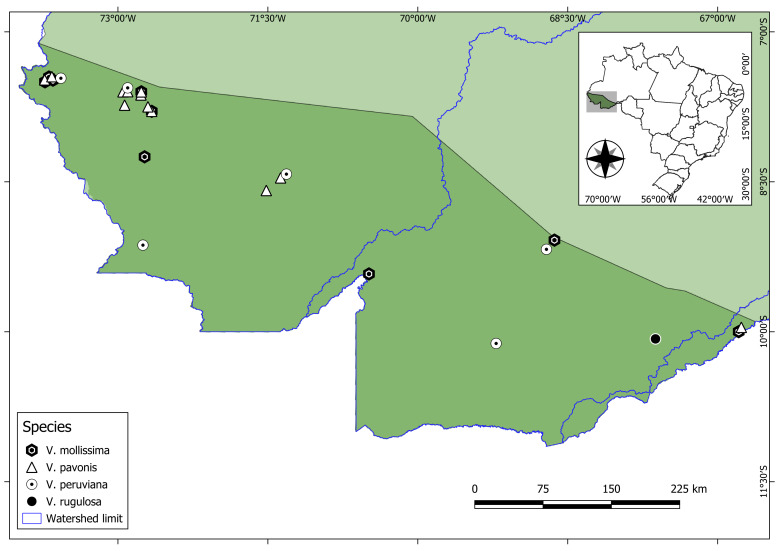
*Virola mollissima*, *V. pavonis*, *V. peruviana* and *V. rugulosa*;

**Figure 4d.**
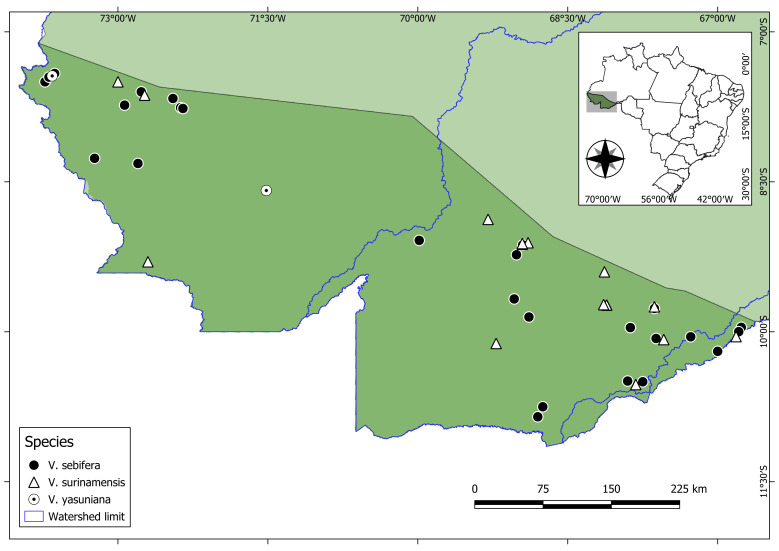
*Virola sebifera, V. surinamensis* and *V. yasuniana*.

**Table 1.** Information of Myristicaceae species recorded in Acre, based on herbaria data. Distribution per watershed, flowering and fruiting months, quantity of records and if they are hyperdominant according to Draper et al. (2021).

**Species**		**Distribution (Juruá and/or Purus Basins)**	**Flowering**	**Fruiting**	**Records**	**Hyperdominant (Yes or No)**
* Compsoneura *	*sprucei*	J	Jan, May, Jun	Sep	7	Y
	*ulei*	J+P	Jan-Mar	May-Jul, Sep, Oct	15	N
* Iryanthera *	*crassifolia*	J	Apr, May	Aug, Oct, Dec	8	Y
	*elliptica*	J	Feb, Apr	Feb	5	Y
	*juruensis*	J+P	Mar-Sep	Mar-May, Aug-Nov	75	Y
	*laevis*	J+P	Apr, Aug	Jul-Nov	16	Y
	*lancifolia*	J	Apr, May, Aug	Feb, Apr, May	15	Y
	*macrophylla*	J	Mar-May	Feb, Oct, Nov	5	Y
	*olacoides*	P	-	Oct	1	N
	*paradoxa*	J	May	Sep, Nov	6	N
	*paraensis*	J	Aug	Feb	2	Y
	*sagotiana*	J	-	Aug, Nov	2	Y
	*tricornis*	J	-	Nov	1	Y
	*ulei*	J	May, Jul, Sep	Mar, Sep, Dec	10	N
* Osteophloeum *	*platyspermum*	J	Sep	Mar, Apr	5	Y
* Otoba *	*glycycarpa*	J	Sep	Sep, Nov	3	Y
	*parvifolia*	J+P	Apr, Jun, Aug-Nov	Jan-Mar, Sep-Dec	32	Y
* Virola *	*albidiflora*	J+P	Nov	Jul	2	N
	*calophylla*	J+P	Feb-May, Nov	Feb, Aug-Oct	25	N
	*carinata*	J+P	Sep, Nov	Nov	5	N
	*decorticans*	J	May	Feb, Dec	4	N
	*duckei*	J+P	Jul, Oct	-	6	Y
	*elongata*	J+P	Jan, Mar-Oct	Jan-Mar, Aug-Dec	48	Y
	*excisa*	J+P	-	Feb, May, Jun, Sep-Nov	12	N
	*flexuosa*	J+P	Sep, Oct	-	4	Y
	*loretensis*	J	May	Sep, Oct	3	N
	*marleneae*	J	-	Aug	1	Y
	*mollissima*	J+P	Nov	Mar, Apr, Sep-Nov	12	N
	*pavonis*	J+P	Feb, Nov	Feb, Jun, Oct-Dec	15	Y
	*peruviana*	J+P	Mar, Jul, Aug, Dec	Jun, Oct, Dec	6	N
	*rugulosa*	P	-	Oct	1	N
	*sebifera*	J+P	Feb-Apr, Jul, Sep-Dec	Apr, Jun-Oct	35	Y
	*surinamensis*	J+P	Apr, May, Jul, Oct, Nov	Mar, Aug-Oct	16	Y
	*yasuniana*	J	Nov	Jun	2	N

**Table 2.** Records by municipality, with the area according to IBGE - Instituto Brasileiro de Geografia e Estatística (2024) and number of specimens collected in CUs or ITs.

**Municipality**	**Area (km²)**	**Records**	**Records in CU or IT**
Acrelândia	1,807	11	0
Assis Brasil	4,974	1	1
Brasiléia	3,916	18	1
Bujari	3,034	17	1
Capixaba	1,701	13	0
Cruzeiro do Sul	8,779	71	5
Epitacolândia	1,654	4	4
Feijó	27,975	1	0
Jordão	5,357	2	1
Mâncio Lima	8,191	87	60
Manoel Urbano	5,453	5	0
Marechal Thaumaturgo	10,633	24	22
Plácido de Castro	1,943	13	0
Porto Acre	6,443	9	0
Porto Walter	8,834	18	6
Rio Branco	3,076	23	7
Rodrigues Alves	6,145	3	0
Santa Rosa do Purus	2,322	2	1
Sena Madureira	23,753	33	8
Senador Guiomard	20,171	14	0
Tarauacá	5,347	29	14
Xapuri	2,604	4	2

**Table 3.** Species occurrence in each watershed in Acre. Bold species in bold have a broad distribution, underlined species have a restricted one.

**Genera**	**Juruá Basin**	**Purus Basin**
* Compsoneura *	***C. ulei***, *C. sprucei*	***C. ulei***
* Iryanthera *	*I. crassifolia*, *I. elliptica*, ***I. juruensis*, *I. laevis***, *I. lancifolia*, *I. macrophylla*, *I. paradoxa*, *I. paraensis, I. sagotiana*, *I. tricornis*, *I. ulei*	***I. juruensis***, ***I. laevis***, *I. olacoides*
* Osteophloeum *	*O. platyspermum*	*-*
* Otoba *	*O. glycycarpa*, ***O. parvifolia***	***O. parvifolia***
* Virola *	***V. albidiflora***, ***V. calophylla***, ***V. carinata***, *V. decorticans*, ***V. duckei***, ***V. elongata***, ***V. excisa**, **V. flexuosa***, *V. loretensis*, *V. marleneae*, ***V. mollissima***, ***V. pavonis***, ***V. peruviana***, ***V. sebifera***, ***V. surinamensis***, *V. yasuniana*	***V. albidiflora***, ***V. calophylla***, ***V. carinata***, ***V. duckei***, ***V. elongata***, ***V. excisa***, ***V. flexuosa***, ***V. mollissima***, ***V. pavonis***, ***V. peruviana***, *V. rugulosa*, ***V. sebifera***, ***V. surinamensis***
**Total**	**32 spp**.	**18 spp**.

**Table 4.** Records of different reproductive structures amongst the treated specimens.

**Reproductive Structure**	**Number of Collections**
Flower bud	8
Staminate flower	124
Pistillate flower	11
Staminate and pistillate flowers	5
Staminate flower and fruit	5
Pistillate flower and fruit	3
Staminate flower, pistillate flower and fruit	1
Fruit	214
Flower undifferentiated	12
Sterile	22
